# French adaptation and validation of the Panic Disorder Severity Scale—self-report

**DOI:** 10.1186/s12888-022-03989-x

**Published:** 2022-06-27

**Authors:** Pasquale Roberge, Patricia Marx, Jonathan Couture, Nathalie Carrier, Annie Benoît, Martin D. Provencher, Martin M. Antony, Peter J. Norton

**Affiliations:** 1 Department of Family Medicine and Emergency Medicine, Université de Sherbrooke, 12th Avenue North, Sherbrooke, QC 3001J1H 5N4 Canada; 2 Centre de Recherche du Centre Hospitalier Universitaire de Sherbrooke (CRCHUS), 12th Avenue North, Sherbrooke, QC 3001J1H 5N4 Canada; 3 École de Psychologie, Pavillon Félix-Antoine-Savard, Université Laval, 2325, rue des Bibliothèques, Québec, QC G1V 0A6 Canada; 4 Department of Psychology, Toronto Metropolitan University, 350 Victoria St, Toronto, ON M5B 2K3 Canada; 5 The Cairnmillar Institute, 391-393 Tooronga Road, Hawthorn East, VIC 3123 Australia

**Keywords:** Assessment scale, Panic disorder, Agoraphobia, Psychometric properties, Cross-cultural validation, Anxiety disorders

## Abstract

**Background:**

The aims of this study were to conduct a cross-cultural validation of the Panic Disorder Severity Scale – Self-Report (PDSS-SR) and to examine psychometric properties of the French-Canadian version.

**Methods:**

A sample of 256 adults were included in the validation study based on data from the baseline interview of a clinical trial on transdiagnostic cognitive-behavioral therapy for mixed anxiety disorders. Participants completed the Anxiety and Related Disorders Interview Schedule (ADIS-5), and self-report instruments including the PDSS-SR, Beck Anxiety Inventory (BAI), Mobility Inventory for Agoraphobia (MIA), Sheehan Disability Scale (SDS), Patient Health Questionnaire (PHQ-9), Social Phobia Inventory (SPIN), Insomnia Severity Index (ISI) and Penn State Worry Questionnaire (PSWQ). The cross-cultural adaptation in French of the PDSS-SR included a rigorous back-translation process, with an expert committee review. Sensitivity to change was also examined with a subgroup of patients (*n* = 72) enrolled in the trial.

**Results:**

The French version of the PDSS-SR demonstrated good psychometric properties. The exploratory factor analysis supported a one factor structure with an eigenvalue > 1 that explained 64.9% of the total variability. The confirmatory factor analysis (CFA) corroborated a one-factor model with a good model fit. Internal consistency analysis showed a .91 Cronbach’s alpha. The convergent validity was adequate with the ADIS-5 clinical severity ratings for panic disorder (*r* = .56) and agoraphobia (*r* = .39), as well as for self-report instruments [BAI (*r* = .63), MIA (accompanied: *r* = .50; alone: *r* = .47) and SDS (*r* = .37)]. With respect to discriminant validity, lower correlations were found with the SPIN (*r* = .17), PSWQ (*r* = .11), ISI (*r* = .19) and PHQ-9 (*r* = .28). The optimal threshold for probable diagnosis was 9 for the PDSS-SR and 4 for the very brief 2-item version. The French version showed good sensitivity to change.

**Conclusions:**

The French version of the PDSS-SR has psychometric properties consistent with the original version and constitutes a valid brief scale to assess the severity of panic disorder and change in severity over time, both in research and clinical practice.

**Supplementary Information:**

The online version contains supplementary material available at 10.1186/s12888-022-03989-x.

Panic disorder (PD) is characterized by recurrent unexpected panic attacks, worry about their consequences, and maladaptive behaviour effects (e.g., avoidance) [1]. Several self-report measures exist for the assessment of distinct characteristics of panic disorder, but few self-report measures can comprehensively assess the severity of panic disorder. The self-report form of the Panic Disorder Severity Scale (PDSS-SR) [2] distinguishes itself as a comprehensive panic disorder severity scale and appears valuable for both clinical practice and research. However, to our knowledge, the PDSS-SR has not yet been adapted and validated in French.

The PDSS-SR was developed as a self-report version of the Panic Disorder Severity Scale (PDSS), a widely used brief clinician-administered instrument for assessing the severity of panic disorder [3]. The PDSS shows adequate psychometric properties with respect to interrater reliability, internal consistency, construct validity and sensitivity to change [3–5]. The PDSS has been translated in multiple languages with validation studies reporting good reliability and validity when compared to the original English version (e.g., [6, 7]). Despite the relevance of this instrument, in some contexts, it is not feasible to train interviewers to administer the PDSS interview, and therefore, Houck et al. [2] developed the self-report version of the PDSS (PDSS-SR). Both versions contain 7 items, and each item is scored from 0 to 4, with increasing values corresponding to increasing severity of panic symptoms [2, 3]. The PDSS-SR assesses the severity of each of 7 dimensions of panic disorder (frequency of panic attacks, distress during panic attacks, anticipatory anxiety, agoraphobic fear/avoidance, interoceptive fear/avoidance, impairment in occupational and social functioning) during the last week on a scale from 0 to 4. A 0 indicates that the patient did not experience the item, and 4 indicates the most severe reaction. This self-report scale has good internal consistency (Cronbach’s α = 0.92), test–retest reliability (ICC = 0.81), interrater reliability, and sensitivity to change [2]. The main difference between the clinician and self-report versions is the period covered by the assessment – the last month and the last week, respectively. The rationale behind this difference was to minimize recall bias for the PDSS-SR and to allow the administration of the questionnaire on a weekly basis to monitor panic symptoms [2]. A validation study conducted with a five-item^1^ version of the instrument, excluding occupational and social interference items, reported moderate reliability and validity [5]. The PDSS-SR has been translated and validated in Chinese, Swedish, Spanish and Korean, and studies reported excellent internal consistency (Cronbach’s α between 0.80 to 0.93), good to excellent correlation with PDSS (*r* = 0.68 to 0.98), good to excellent test–retest reliability (ICC between 0.65 to 0.96), adequate convergent and discriminant validity, and a significant sensitivity to change [6, 8–10]. However, there was dissimilarity in the factor structure of the PDSS-SR, with the Korean version supporting a unidimensional structure [8], the Chinese and Spanish versions supporting a two-factor model [9, 10], and the Swedish version yielding inconclusive results [6]. Recent studies have also suggested thresholds for probable panic disorder with the PDSS-SR [9] and a very brief 2-item version focusing solely on distress during panic attacks and agoraphobic avoidance [11].

The goal of our study was to conduct a cross-cultural adaptation and validation of the PDSS-SR for the assessment of panic disorder with a French-Canadian sample of adults with anxiety symptoms taking part in a clinical assessment to enrol in a randomized controlled trial of transdiagnostic group cognitive-behavioral therapy. The specific objectives of the study were to: 1) describe the validity and reliability of the French-Canadian adaptation of the PDSS-SR; 2) explore the optimal cut-off score for probable diagnosis; 3) examine sensitivity to change with a subset of patients meeting DSM-5 criteria for panic disorder enrolled in the clinical trial.

## Methods

### Participants

Data were drawn from the baseline assessment (T0) and posttreatment assessment (T1) of participants to a pragmatic clinical multicentre trial of transdiagnostic group cognitive-behavioral therapy (CBT) for anxiety disorders conducted in Quebec, Canada. The study was conducted in three integrated health and social services centers, comprising Quebec City and adjacent areas (i.e., urban and rural population of 737 000 inhabitants); Estrie (i.e., urban and rural population of 474 000 inhabitants); and Laval City (i.e., predominantly urban population of 435 000 inhabitants). Complete methodology and primary outcome results for the trial were described previously [12]. The trial inclusion criteria comprised the following: (1) ages 18 to 65, (2) fluent in spoken and written French; (3) DSM-5 diagnostic criteria met for at least one of the following principal anxiety disorders: Panic Disorder, Agoraphobia, Generalized Anxiety Disorder and/or Social Anxiety Disorder with a clinical severity rating (CSR) ≥ 4 on the Anxiety and Related Disorders Interview Schedule for DSM-5 (ADIS-5) [13]. The trial exclusion criteria comprised: (1) active suicidal intentions, psychosis, bipolar disorder, and active substance-related and addictive disorder in the past 12 months; (2) marked cognitive impairment; (3) consultation with a psychiatrist in the past 12 months.

A three-stage process was used for patient recruitment. First, we advertised the study in the general population and community-based care settings through regional newspapers, geo-located *Facebook* and *Google AdWords* publicity, and bulletin boards*.* Self-referred individuals completed a brief screening online survey that included the Generalized Anxiety Disorder-7 (GAD-7; cut point value ≥ 8 for anxiety disorders) [14], and other measures. Second, clinical evaluators completed a brief telephone-screening interview with potential participants. Third, face-to-face clinical assessment interviews were conducted with potential candidates to assess their eligibility based on the ADIS-5 assessment (T0). All participants taking part in the third stage of assessment (T0) of the clinical trial and returning baseline questionnaires, with a principal anxiety disorder, regardless of being enrolled in the trial, were potentially eligible for the secondary validation study. The sample for objectives 1 and 2 was composed of all T0 participants with a complete dataset. The sample for objective 3 was composed of participants enrolled in the clinical trial, meeting DSM-5 criteria for panic disorder and with a complete pre-post dataset.

The study was approved by the principal ethics review board (Comité d’éthique de la recherche du Centre intégré universitaire de santé et de services sociaux de l’Estrie – Centre hospitalier universitaire de Sherbrooke, #MP-22–2016-570) and the boards of the other institutions (Comité d’éthique de la recherche de l’Institut universitaire en santé mentale de Quebec, #2017–166; Comité scientifique et d’éthique de la recherche – CISSS de Laval, #2016–2017 / C54). All patients gave their written informed consent.

### Intervention

The experimental condition was a transdiagnostic group cognitive-behavioral therapy program that comprised education and self-monitoring, specific cognitive restructuring, graduated exposure and response prevention, and generalized cognitive restructuring [15]. The intervention was delivered to groups of 8–10 patients over a 12-week period by two psychologists or psychotherapists. The treatment-as-usual (TAU) control group continued receiving usual care with no restrictions throughout the trial.

### Measures

The initial in-person assessment interview (T0; pre-random assignment) comprised the consent form, administration of the ADIS-5 and a questionnaire on sociodemographic data, health care costs, medication, and work performance by a trained PhD level evaluator. Patients were also given a participant’s booklet with the self-report questionnaires, and the same booklet was used at T0 and T1. Only measures used for the secondary validation study are described here.

#### Diagnostic interview

The ADIS-5 was used to assess DSM-5 diagnostic criteria for anxiety disorders and comorbid mental disorders [13]. A significant clinical improvement at posttreatment for panic disorder was defined as a clinical severity rating (CSR) < 4 or at least a 2-points decrease on the 8-point scale.

#### PDSS-SR translation

To conduct a cross-cultural translation of the PDSS-SR [2] in French for Canada, we followed previously published guidelines [16, 17]. Two forward translations of the original English PDSS-SR were performed, including one by a professional translator and the other by a research assistant. The rationale to having two forward translations is that it allows both translations to be compared, permitting the identification of translation errors and varied interpretations of phrasing of items in the source language [17]. The two translated questionnaires were then reconciled into one French-Canadian version through a consensus discussion. The process of reconciliation allowed any differences between forward translations to be compared and the preferred phrasing was integrated into the final version. Minor differences were observed in the translation but were easily solved by an expert committee composed of the research team and collaborators involved in the translation process. Additionally, this process minimizes the chances that the final French-Canadian version could be biased toward one translator’s writing style [17]. Following this, two back translations were carried out by fluent English speakers to ensure that all questions retained their original meaning when returned to the source language. These back translations were compared to the original English version of the PDSS-SR, and minor revisions of the French phrasing were conducted to ensure conceptual equivalence of all items. The instrument’s instructions, questions, and response options were reviewed to ensure synonyms and expressions had similar meanings and were culturally equivalent, to solve any remaining grammatical difficulties, and assert reading level adequacy.

#### Other self-report questionnaires

The patient's booklet comprised instruments with good psychometric properties and validated in French. Measures of anxiety symptoms included the *Beck Anxiety Inventory* (BAI) [18, 19], the *Mobility Inventory for Agoraphobia* (MIA) [20, 21], the *Social Phobia Inventory* (SPIN) [22, 23] and the *Penn State Worry Questionnaire* (PSWQ) [24, 25]. Depressive symptoms were assessed with the *Patient Health Questionnaire* (PHQ-9) [26, 27]. The *Sheehan Disability Scale* (SDS) was used as a measure of disability in work, social life and family life [28]. The *Insomnia Severity Index* (ISI) was also administered to examine the severity of insomnia and associated difficulties [29, 30]. Only the self-reported questionnaires used for this validation study were listed, but other were used in the main study.

### Procedure

#### Data analyses

The factor structure of the PDSS-SR was examined with exploratory factor analysis (EFA) and with confirmatory factor analysis (CFA). For the EFA, Promax rotation was used, factors with eigenvalues > 1 were retained and a factor loading higher of 0.40 was retained to explain the target factor. A first analysis was performed without imposing any factor and a second by imposing two factors according to previous research. Then, CFA with one-factor was used to examine the fitness of the structure obtained with EFA. The diagonally weighted least squares estimation method was used since it was more appropriate for ordinal data [31]. The model fit was assessed with standardized root mean square residual (SRMR), adjusted goodness of fit index (AGFI) and normed fit index (NFI). A SRMR below 0.08, AGFI higher 0.95 and NFI higher 0.95 indicate a good model fit [32].

Internal consistency of the French-Canadian PDSS-SR was assessed using Cronbach’s α. Convergent and discriminant validity were examined with Spearman correlations as several variables were not normally distributed [33]. The correlations were assessed for the total score and items of the PDSS-SR with ADIS-5 CSR for anxiety and depressive disorders, as well as different self-report questionnaires. Based on the concept of panic disorder and previous studies [5, 6, 8, 10], this allowed for the examination of convergent validity with another measure of panic disorder (ADIS-5) and related agoraphobia (ADIS-5, MIA), of anxiety symptoms (BAI), as well as disability (SDS). For discriminant validity, lower correlations were expected with other questionnaires that measure different constructs (ADIS-5, ISI, PHQ-9, SPIN, PSWQ), although comorbidity would need to be considered in the interpretation of findings. Receiver operating characteristic (ROC) curves for the PDSS-SR were computed to evaluate the performance of the score to distinguish participants with and without panic disorder with the PDSS-SR full scale. We also conducted an exploratory analysis of the 2-item version recommended by Forsell et al. [11]. The Youden index was used to define the best cut-offs.

To examine sensitivity to change, a linear mixed regression model was performed with the maximum-likelihood method and the variance components correlation matrix on the PDSS-SR considering the intervention group, time and interaction (evolution between groups). The intra-group and intra-site variability of therapy was controlled with a random effect on the participants nested in the therapy groups, nested in the three sites. To control for the variability between groups, a second random effect on the therapy groups nested in the three sites was added. Since the residuals were normally distributed, bootstrap resampling was not necessary. Sensitivity to change was analysed for two scenarios of improvement at baseline (T0) and posttreatment (T1) assessment periods in participants with panic disorder enrolled in the clinical trial, including the comparison of experimental and active control conditions and the comparison of participants with significant clinical improvement for panic disorder based on ADIS-5 CSR score for panic disorder. CFA analyses were performed with SAS version 9, other analyses were performed with IBM SPSS Statistics version 25. GraphPad Prism version 7 was used to create the figures.

## Results

### Participants

A complete baseline dataset was available for 256 participants, including sociodemographic data, ADIS-5 interview and self-report questionnaires. Table 1 shows the sociodemographic and clinical characteristics of the sample. Participants were mostly women (85.2%), and the mean age was 36.9 years (SD = 12.4, range = 18–79). The majority of participants had at least a postsecondary degree (88.9%) and 52.9% were married or living with a partner. The sample was mostly composed of participants with comorbid anxiety and related disorders, with DSM-5 criteria being met for generalized anxiety disorder (77.7%), social anxiety disorder (61.7%), panic disorder (40.6%), agoraphobia (29.3%), and depression (23.8%).

**Table 1:** Demographic and clinical characteristics of the study sample

Features	Total^a^ (*n* = 256)
Age (years), mean (*SD*)	36.9 (12.4)
Women, n (%)	218 (85.2)
Education, n (%)	
Secondary or less	28 (11.0)
Postsecondary/Vocational	121 (47.6)
University	105 (41.3)
Marital status, n (%)	
Married/living with a partner	135 (52.9)
Single	100 (39.2)
Divorced/separated	20 (7.8)
Occupation, n (%)	
Work full time	153 (59.8)
Student	50 (19.5)
Work part time	20 (7.8)
Unemployment	23 (9.0)
Retirement	10 (3.9)
Panic disorder	104 (40.6)
Other anxiety disorder ^b^	246 (96.1)
Generalized anxiety disorder	199 (77.7)
Social anxiety disorder	158 (61.7)
Agoraphobia	75 (29.3)
Specific phobia	43 (16.8)
Obsessive Compulsive Disorder	15 (5.9)
Posttraumatic stress disorder	9 (3.5)
Alcohol or substance disorder	1 (0.4)
Depression	61 (23.8)

^a^There were two missing data for education (*n* = 254), and one missing data for marital status (*n* = 255)

^b^Other anxiety disorder was defined by presence of agoraphobia, social anxiety disorder, specific phobia or generalized anxiety disorder

### Factor structure and confirmatory factor analysis

The EFA had only one eigenvalue > 1 for a one-factor structure, explaining 64.9% of the total variability, and each item was well represented (eigenvalue 4.54; factor loadings = 0.77 to 0.83) (Table 2). The eigenvalue of the second factors was 0.77 and explained 11.0% of the total variability. These results suggested a one-factor structure. As several authors in the literature reported a two-factor structure, we also explored this possibility. By repeating the exploratory analysis with two imposed factors, we observed that items 1, 2 and 3 were well represented on the second factor with factor loading > 0.40, and that items 4 to 7 were well represented on the first factor. A CFA was subsequently performed and confirmed a one-factor model with all fit indices indicating a good model fit (SRMR = 0.0602, AGFI = 0.9842 and NFI = 0.9859). Supplementary Fig. 1 presents factor loading of CFA in a path diagram. No two-factor structure CFA was performed.

**Table 2:** Component matrix from exploratory factor analysis

	One-factor structure	Two-factor structure	
	Factor 1	Factor 1	Factor 2
1. Panic attack frequency	0.79	-0.06	0.96
2. Distress	0.81	-0.01	0.94
3. Severity of anticipatory anxiety	0.77	0.36	0.50
4. Agoraphobic fear/avoidance	0.82	0.86	0.01
5. Fear/avoidance of panic-related sensations	0.80	0.91	-0.07
6. Work impairment	0.83	0.73	0.17
7. Social impairment	0.82	0.87	-0.01

### Internal consistency

Cronbach’s α for the French-Canadian PDSS-SR was 0.91. Mean score for French PDSS-SR was 8.73 ± 6.20. Mean scores and standard deviations for individual items, corrected item-total correlations, Cronbach’s α by omitting items from the scale and correlations between items are presented in Table 3. Corrected item-total correlations range between 0.69 to 0.76, indicating a good relationship with the PDSS-SR when the item was excluded.

**Table 3:** Cronbach's α, correlations between each item and the total score, and correlations between items and the French PDSS-SR

	Score Mean ± SD	Corrected item-total correlations	Cronbach’s α if item deleted	Correlations between items						
				1. Panic attack frequency	2. Distress	3. Severity of anticipatory anxiety	4. Agoraphobic fear/avoidance	5. Fear/avoidance of panic-related sensations	6. Work impairment	7. Social impairment
1. Panic attack frequency	1.34 ± 1.02	0.71	0.90	1						
2. Distress	1.45 ± 1.17	0.73	0.90	0.79	1					
3. Severity of anticipatory anxiety	1.33 ± 1.12	0.69	0.90	0.56	0.61	1				
4. Agoraphobic fear/avoidance	1.11 ± 1.11	0.74	0.89	0.52	0.53	0.61	1			
5. Fear/avoidance of panic-related sensations	1.03 ± 1.02	0.72	0.90	0.49	0.52	0.52	0.69	1		
6. Work impairment	1.22 ± 1.17	0.76	0.89	0.58	0.59	0.54	0.62	0.64	1	
7. Social impairment	1.25 ± 1.08	0.74	0.89	0.51	0.54	0.55	0.65	0.63	0.71	1

### Convergent and discriminant validity

Table 4 shows the correlation coefficients between the French-Canadian PDSS-SR, ADIS-5 and other self-report measures. Spearman correlations were used for all variables as PDSS-SR and ADIS-5 were not normally distributed. For the ADIS-5 CSR, there was convergent validity with panic disorder (*r* = 0.56) and agoraphobia (*r* = 0.39), with no significant correlation observed for other anxiety and related disorders, except generalized anxiety disorder (*r* = 0.13). Correlations for each of the seven items with ADIS-5 CSR for panic disorder ranged from *r* = 0.40 for distress to *r* = 0.55 for severity of anticipatory anxiety. Item correlations with ADIS-5 CSR for agoraphobia ranged from *r* = 0.24 for distress to *r* = 0.46 for agoraphobic fear and avoidance. For other ADIS-5 CSR diagnosis, there were no item correlations over *r* = 0.21 (i.e., work impairment correlated with generalized anxiety disorder).

**Table 4:** Correlation between French PDSS-SR, ADIS-5 and other self-report measures

	n	PDSS-SR	1. Panic attack frequency	2.Distress	3. Severity of anticipatory anxiety	4. Agoraphobic fear/avoidance	5.Fear/avoidance of panic-related sensations	6. Work impairment	7. Social impairment
Anxiety and Related Disorders Interview Schedule for DSM-5 Clinician Severity Rating									
Panic disorder	256	0.56 ***	0.43 **	0.40 ***	0.55 ***	0.53 ***	0.41 ***	0.43 ***	0.42 ***
Agoraphobia	256	0.39 ***	0.27 **	0.24 ***	0.37 ***	0.46 ***	0.37 ***	0.25 ***	0.25 ***
Depression	256	0.08	0.08	0.08	0.07	0.02	0.05	0.09	0.06
Social anxiety disorder	256	-0.01	-0.06	-0.03	-0.08	0.02	-0.06	0.01	0.14 *
Specific phobia	256	0.11	0.08	0.13 *	0.09	0.04	0.12	0.09	0.08
Generalized anxiety disorder	256	0.13 *	0.08	0.06	0.03	0.04	0.10	0.21 ***	0.12 *
Posttraumatic stress disorder	256	0.06	-0.01	0.02	-0.03	0.10	0.09	0.08	0.06
Obsessive compulsive disorder	256	0.07	0.06	0.07	0.07	0.03	0.01	0.04	0.08
Self-report measures									
Beck Anxiety Inventory	256	0.63 ***	0.53 ***	0.53 ***	0.49 ***	0.52 ***	0.44 ***	0.50 ***	0.52 ***
Mobility Inventory for Agoraphobia, accompanied	235	0.50 ***	0.31 ***	0.29 ***	0.39 ***	0.50 ***	0.47 ***	0.44 ***	0.48 ***
Mobility Inventory for Agoraphobia, alone	232	0.47 ***	0.32 ***	0.27 ***	0.35 ***	0.49 ***	0.41 ***	0.45 ***	0.42 ***
Sheehan Disability Scale	249	0.37 ***	0.21 ***	0.26 ***	0.22 ***	0.26 ***	0.30 ***	0.46 ***	0.40 ***
Patient Health Questionnaire	256	0.28 ***	0.21 ***	0.26 ***	0.15 *	0.17 **	0.26 ***	0.30 ***	0.22 ***
Social Phobia Inventory	252	0.17 **	0.11	0.11	0.09	0.16 **	0.10	0.17 **	0.26 ***
Insomnia Severity Index	254	0.19 **	0.15 *	0.17 **	0.06	0.05	0.19 **	0.23 ***	0.19 **
Penn State Worry Questionnaire	256	0.11	0.09	0.12	0.07	0.01	0.09	0.13 *	0.08

^*^p < .05, **p < .01; ***p < .001

Considering convergent validity based on self-report measures, the PDSS-SR was more correlated with BAI (*r* = 0.63) and MIA (with a companion: *r* = 0.50, alone: *r* = 0.47) than with SDS (*r* = 0.37). As the correlation with the PHQ-9 was low (*r* = 0.28), but significant, a test of dependent correlation was computed by comparing the correlation between PDSS-SR, BAI and PHQ-9. Results showed that the PDSS-SR was more correlated with the BAI than the PHQ-9 (z = 5.11, *p* < 0.001). Moreover, the correlation between PDSS-SR and PHQ-9 was not significant after adjusting for SDS with partial correlations (*r* = 0.10, *p* = 0.11). For discriminant validity with other self-report measures, the correlation was lower with SPIN (*r* = 0.17), PSWQ (*r* = 0.11) and ISI (*r* = 0.19). PDSS-SR items were predominantly correlated with the BAI (*r* = 0.44 to *r* = 0.53), the MIA accompanied and alone (*r* = 0.27 to *r* = 0.50) and the SDS (*r* = 0.21 to *r* = 0.46) than with other measures.

### Sensitivity and specificity of cut-off scores

**Fig. 1:**
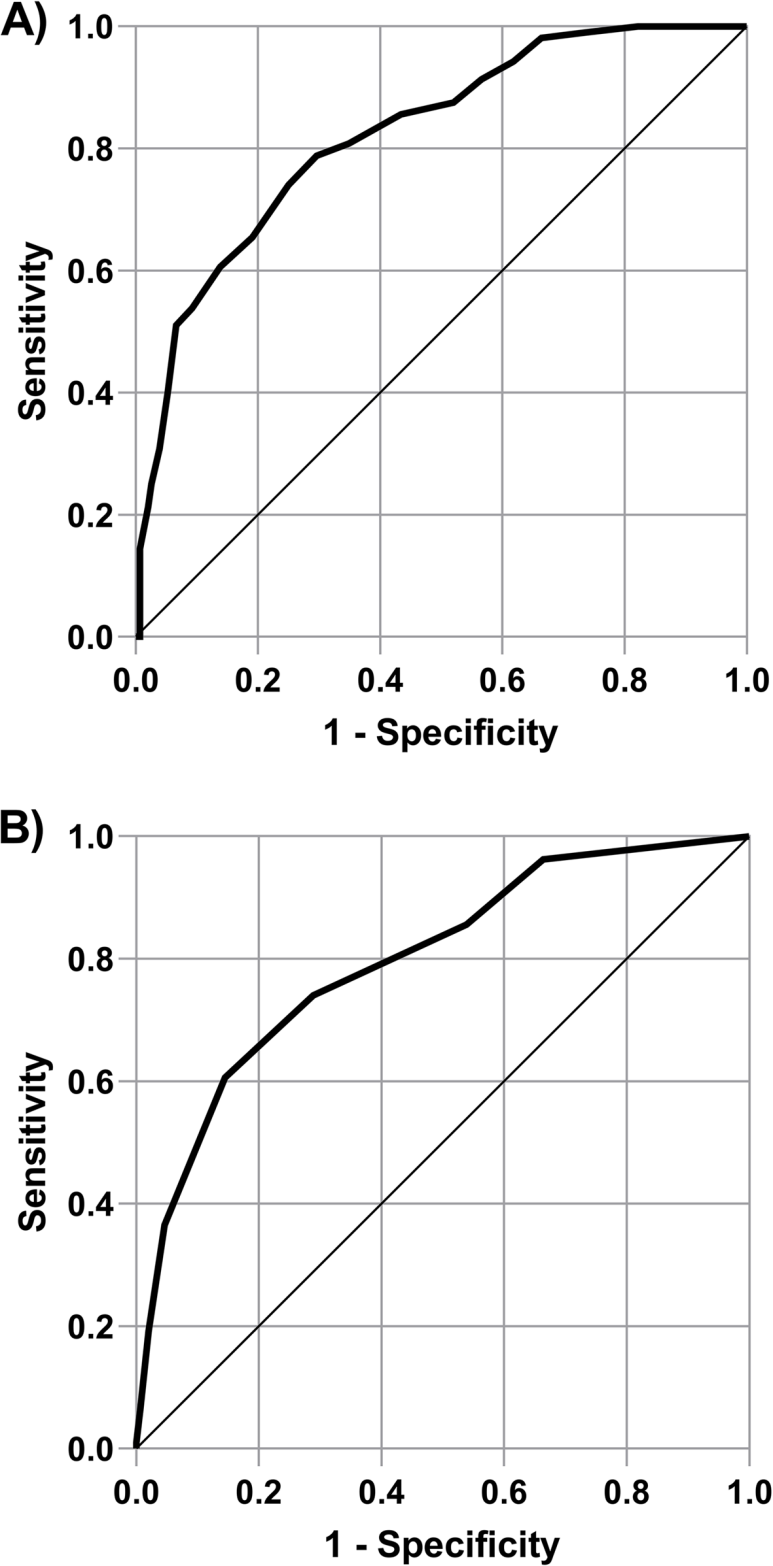
Receiver-operating characteristic (ROC) curve for the PDSS-SR (**A**) and brief PDSS-SR scale (**B**)

A ROC curve analysis was performed to detect participants for whom DSM-5 criteria were met for panic disorder within our sample, based on the PDSS-SR score (see Fig. 1). The area under the curve (AUC) was 0.82 (95% CI 0.77-0.87; *p* < 0.001). The Youden index indicated that the optimal threshold was 9 with a sensitivity of 78.8% and a specificity of 70.4%. In our sample, this corresponds to a positive predictive value (PPV) of 64.6% and a negative predictive value (NPV) of 82.9%. The Supplementary Table 1 presents sensitivity, specificity, Youden index, PPV and NPV for each cut-point for the PDSS-SR.

A ROC curve analysis was also performed to detect panic disorder based on the 2-item PDSS-SR composed of items 2 (i.e., distress during panic attacks) and 4 (i.e., agoraphobic avoidance) [11]. The correlation with the original scale was *r* = 0.93 and the AUC was 0.80 (95% CI 0.74-0.85; *p* < 0.001). The best cut-off for the very brief scale was 4, with a sensitivity of 60.6% and a specificity of 85.5%.

### Sensitivity to change

**Fig. 2:**
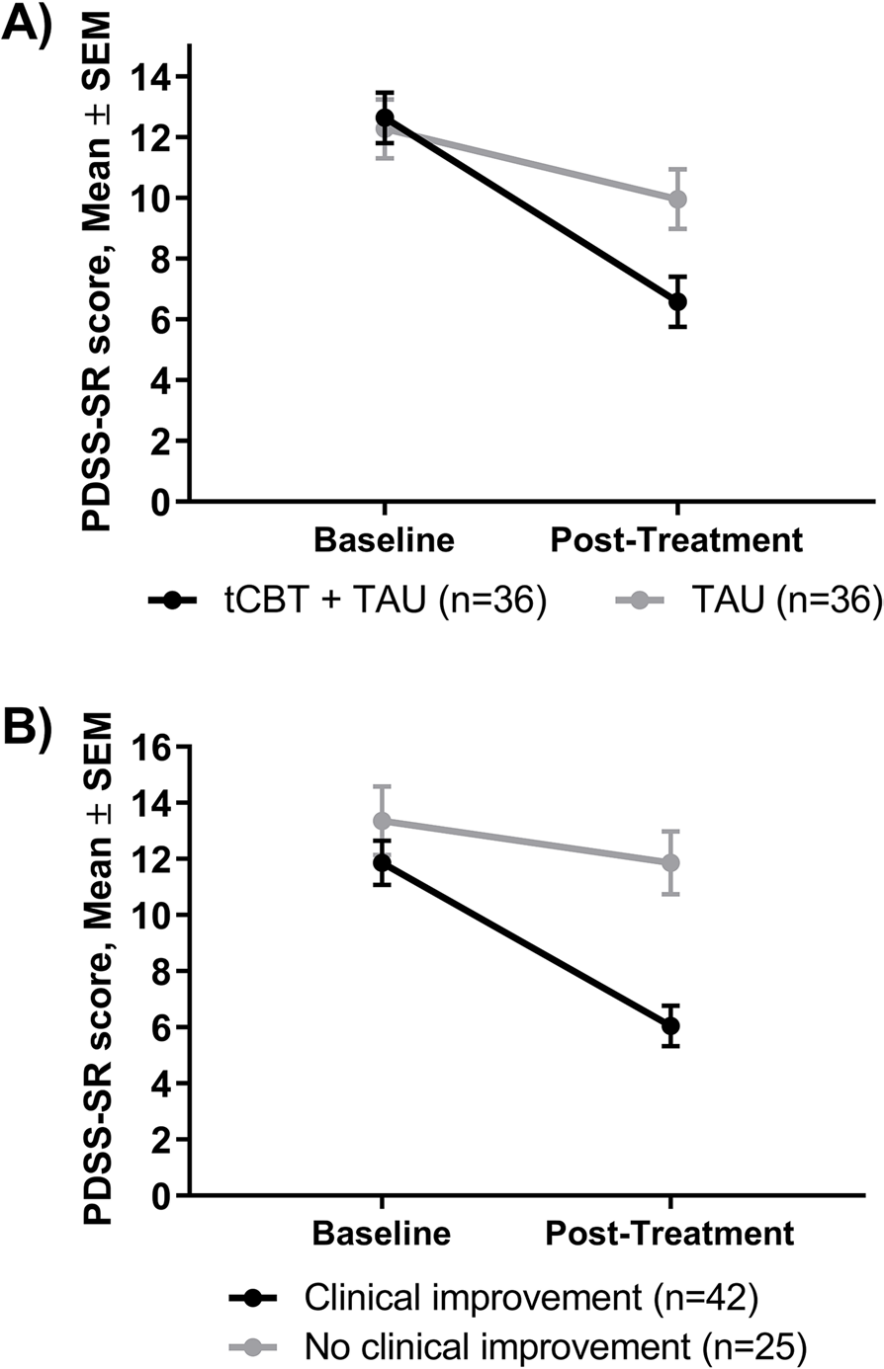
Sensitivity to change according to experimental group in the clinical trial (**A**) or clinical improvement (**B**). tCBT: Transdiagnostic group cognitive-behavioral therapy; TAU: Treatment-as-usual

We completed the sensitivity analysis with a subgroup of 72 patients for whom ADIS-5 criteria were met for panic disorder at baseline, and who were enrolled in the trial with a complete dataset at T0 and T1 (Fig. 2). Two definitions of clinical improvement were examined. First, we conducted a comparative analysis between participants randomized to the intervention condition (*n* = 36) compared to participants in the TAU only active control condition (*n* = 36). At T0, there was no difference in mean (*SD*) score for PDSS-SR in intervention and control conditions respectively (12.6 ± 5.0 vs 12.3 ± 5.8; *p* = 0.777). Results of the linear mixed model indicated a significant difference in time (*p* = 0.009) and a significant difference for the interaction (difference between group of 3.74; 95% CI 1.29–6.18, *p* = 0.003), suggesting that the decrease in PDSS-SR score over time was significantly larger in the intervention condition. Second, we categorized participants (*n* = 67) based on clinical outcomes as an indicator of significant clinical improvement. Overall, 62.7% of participants (*n* = 42) met criteria for clinical improvement based on an ADIS CSR score < 4 or a decrease of at least 2 points. From the linear mixed model, no significant difference was observed in time or in group, but the interaction was significant, with a larger decrease in PDSS-SR score over time in the intervention condition (difference between group of 4.31; 95% CI 1.63–6.99, *p* = 0.002).

## Discussion

We conducted a cross-cultural translation and validation of the PDSS-SR for French-Canadians based on secondary data from a clinical trial on transdiagnostic group cognitive-behavioral therapy for anxiety disorders in adults. Data for the validation study were drawn from the initial assessment of potential participants with anxiety symptoms interested in enrolling in the trial and from a subset of participants with panic disorder enrolled in the trial with a complete dataset at posttreatment to examine sensitivity to change. The results obtained are essentially similar to those reported for the original English language version of the PDSS-SR [2] and from subsequent validation studies [5, 6, 8–10], which support the relevance of this self-report instrument in measuring the severity of panic disorder.

The factor structure of the PDSS-SR is not consistent across studies. The EFA and CFA suggested a one-factor structure well represented by all the seven items, with 65.5% of the variability explained by this factor, that was consistent with Lee et al. [8]. Santacana et al. [10] reported a two-factor structure (items 1 and 2,items 3 to 7). Liu et al. [9] also obtained a closely related two-factor loading (items 1 to 3,items 4 to 7). Svensson et al. [6] were more cautious in stating that the CFA did not confirm a two-factor structure, but the EFA suggested a two-factor structure. While our study does not resolve the issue of the factor structure for the PDSS-SR, it complements previous studies with a heterogenous sample of participants with diverse anxiety and related disorders, and with extensive comorbidity.

The results of the validation showed excellent internal consistency, with moderate to high correlations between items (i.e., 0.49 to 0.79), and the deletion of any items would not improve the Cronbach’s alpha. The mean PDSS-SR score was comparable to the initial study’s mean score of 9.0 (SD = 6.6) with a mixed sample of outpatients [2], and lower than in studies conducted exclusively with patients meeting panic disorder criteria, ranging from 10.49 (SD = 4.58) in the Chinese sample [9], to 10.82 (SD = 5.06) in the Korean version [8], to 12.43 (SD = 4.51) in the Swedish version [6] and 12.57 (SD = 5.33) in the Spanish version [10].

Our results demonstrate good construct validity for the French-Canadian PDSS-SR, with the scale correlating significantly with the clinician-reported ADIS-5 CSR for panic disorder and agoraphobia, but not with other anxiety and related disorders. For self-report measures of anxiety, the highest correlations were observed for the general measure of anxiety (i.e., BAI), agoraphobia (i.e., MIA) and disability (i.e., SDS), but significant correlations were also noted for symptoms of social anxiety disorder (i.e., SPIN), insomnia (i.e., ISI) and depression (i.e., PHQ-9). This was expected due to high rates of comorbidity in our mixed sample of participants with anxiety and depressive symptoms. In other validation studies, the examination of the same concepts led to overall moderate to large significant correlations with diverse self-report instruments, and variations in range were expected considering the clinical characteristics of each sample in terms of principal diagnosis, comorbidity and severity. In the Lee et al. (2009) [8] study based on a sample of patients with principal diagnoses of panic disorder, correlations with general anxiety with the BAI were similar, but were higher for agoraphobia and depression. Svensson et al. [6] also reported a similar correlation with agoraphobia, but a higher correlation with depression. Wuyek et al. [5] reported lower range correlations with self-report agoraphobia *(r* = 0.27-0.29), which could perhaps be related to the exclusion of interference items of the scale. Also of interest, the correlation with disability (*r* = 0.37) was lower than for the other studies using the same self-report measure with respectively *r* = 0.77 and *r* = 0.63 [6, 10]. To explore the impact of a predominantly panic disorder sample on correlations with disability, we conducted a subgroup analysis with participants for whom DSM-5 criteria for panic disorder were met in our sample, and the correlation with disability increased to *r* = 0.56.

We examined the sensitivity and specificity of the 7-item PDSS-SR as a potential screener for panic disorder. The optimal statistical cut-off to identify panic disorder in our heterogenous sample was 9 according to the ROC curve, with higher sensitivity (78.8%) and specificity (70.4%), which is same cut-point reported by Forsell et al. [11]. However, it is in contrast with the cut-point of 4 identified by Liu et al. [9] that presented high sensitivity (96.0%) but lower specificity (61.3%). Choosing the optimal threshold is challenging and depends on the research or clinical goal of the assessment, regarding the potential consequences of false positive and false negative classification. The dissimilar curve profiles may be related to the comparison group without panic disorder in both studies, as Liu et al. [9] included patients with other anxiety disorders and also “healthy subjects.” The examination of cut-points applicable in clinical and research settings is a key emergent area of psychometric research with the PDSS-SR, and we provided a supplementary file with sensitivity, specificity, PPV and NPV to support decision making and comparison with other studies to clarify optimal cut-points. For the exploratory examination of the very brief PDSS-SR for screening, the optimal cut-point was determined as 4, and specificity was high (85.5%), but the sensitivity of the scale was low (60.7%). As a comparison point, with the cut-off of 3 established by Forsell et al. [11], the sensitivity was higher (74.0%) but the specificity was lower (71.1%), which may be preferable in a brief screening context.

Finally, we examined whether the PDSS-SR was sensitive to change with a subsample of participants enrolled in the clinical trial for whom panic disorder diagnostic criteria were met based on the ADIS-5, and our results are consistent with previous studies [8, 10]. They support sensitivity to change of the PDSS-SR when comparing the transdiagnostic group CBT condition and active control condition, as well as with a classification based on significant clinical improvement.

Among the strengths of the study, we conducted a rigorous adaptation and validation of the PDSS-SR with an heterogenous sample of participants seeking care for anxiety disorders, with a range of principal and additional anxiety and related disorders, severity, and psychiatric comorbidity. These findings complement previous validation studies focusing specifically on panic disorder. The convergent validity of the PDSS-SR was demonstrated with the clinician-administered ADIS-5 according to DSM-5 diagnostic criteria, an analysis not yet reported in the available literature. Shear et al. [3] had initially demonstrated convergent validity between the clinician-administered PDSS and panic disorder diagnoses with DSM-III-R. The clinical trial also allowed for the examination of sensitivity to change with clinician-reported and self-report measures. Among limitations to consider in the interpretation of the results, the study design did not allow for temporal stability assessment and the sociodemographic characteristics of the sample may limit generalization, with a predominant sample of women and over 40% having a university degree.

## Conclusion

Our data indicate that the French-Canadian PDSS-SR has comparable psychometrics properties to the original version of the PDSS-SR and provide further support for sensitivity to change as well as for the use of the instrument as a screener with a cut-off value. These are key considerations to encourage the widespread use of the PDSS-SR. The PDSS-SR is brief and closely aligned with DSM-5 diagnostic criteria for panic disorder, much like the widely used PHQ-9 for depression. The assessment efficacy and brevity of the PDSS-SR for panic disorder provides support to increased use in research and clinical practice to screen for panic disorder and to assess diagnostic-specific clinical progress over time. Furthermore, while global measures of anxiety symptomatology are often used in epidemiological surveys, clinical trials and mental health monitoring, the PDSS-SR contributes to the perspective that efficient diagnosis-specific measurement for anxiety disorders is also attainable at the population level.

## Supplementary Information

**Additional file 1: Supplementary Table 1. Supplementary Figure 1. **Path diagram from confirmatory factor analysis.

## Data Availability

The datasets used and/or analysed during the current study are available from the corresponding author on reasonable request.

## Abbreviations

ADIS-5: Anxiety and Related Disorders Interview Schedule for DSM-5
AGFI: Adjusted Goodness of Fit Index
AUC: Area Under the Curve
BAI: Beck Anxiety Inventory
CBT: Cognitive-Behavioral Therapy
CFA: Confirmatory Factor Analysis
CSR: Clinical Severity Rating
EFA: Exploratory Factor Analysis
GAD-7: Generalized Anxiety Disorder-7
ISI: Insomnia Severity Index
MIA: Mobility Inventory for Agoraphobia
NFI: Normed Fit Index
NPV: Negative Predictive Value
PDSS: Panic Disorder Severity Scale
PDSS-SR: Panic Disorder Severity Scale – Self-Report
PHQ-9: Patient Health Questionnaire
PPV: Positive Predictive Value
PSWQ: Penn State Worry Questionnaire
ROC: Receiver Operating Characteristic
SDS: Sheehan Disability Scale
SPIN: Social Phobia Inventory
SRMR: Standardized Root Mean Square Residual

## Footnotes

1 The version of the PDSS-SR used by Wuyek et al. [5] included only five items because of a clerical error (personal communication, Martin M. Antony). The correct version of the PDSS-SR includes seven items.

## References

[1] American Psychiatric Association (2013). Diagnostic and statistical manual of mental disorders.

[2] Houck PR, Spiegel DA, Shear MK, Rucci P (2002). Reliability of the self-report version of the panic disorder severity scale. Depress Anxiety.

[3] Shear MK, Brown TA, Barlow DH, Money R, Sholomskas DE, Woods SW, Gorman JM, Papp LA (1997). Multicenter collaborative panic disorder severity scale. Am J Psychiatry.

[4] Furukawa TA, Katherine Shear M, Barlow DH, Gorman JM, Woods SW, Money R, Etschel E, Engel RR, Leucht S (2009). Evidence-based guidelines for interpretation of the Panic Disorder Severity Scale. Depress Anxiety.

[5] Wuyek LA, Antony MM, McCabe RE (2011). Psychometric properties of the panic disorder severity scale: clinician-administered and self-report versions. Clin Psychol Psychother.

[6] Svensson M, Nilsson T, Johansson H, Viborg G, Perrin S, Sandell R (2019). Psychometric analysis of the Swedish panic disorder severity scale and its self-report version. Nord J Psychiatry.

[7] Yamamoto I, Nakano Y, Watanabe N, Noda Y, Furukawa TA, Kanai T, Takashio O, Koda R, Otsubo T, Kamijima K (2004). Cross-cultural evaluation of the Panic Disorder Severity Scale in Japan. Depress Anxiety.

[8] Lee EH, Kim JH, Yu BH (2009). Reliability and validity of the self-report version of the Panic Disorder Severity Scale in Korea. Depress Anxiety.

[9] Liu X, Xu T, Chen D, Yang C, Wang P, Huang X, Cheng W, Shen Y, Liu Q, Wang Z (2020). Reliability, validity and cut-off score of the Chinese version of the panic disorder severity scale self-report form in patients with panic disorder. BMC Psychiatry.

[10] Santacana M, Fullana MA, Bonillo A, Morales M, Montoro M, Rosado S, Guillamat R, Valles V, Perez V, Bulbena A (2014). Psychometric properties of the Spanish self-report version of the panic disorder severity scale. Compr Psychiatry.

[11] Forsell E, Kraepelien M, Blom K, Isacsson N, Jernelov S, Svanborg C, Rosen A, Kaldo V (2019). Development of a very brief scale for detecting and measuring panic disorder using two items from the Panic Disorder Severity Scale-Self Report. J Affect Disord.

[12] Roberge, P., Provencher, M. D., Gaboury, I., Gosselin, P., Vasiliadis, H.-M., Benoît, A., Carrier, N., Antony, M. M., Chaillet, N., Houle, J., Hudon, C., & Norton, P. J. (2020). Group transdiagnostic cognitive-behavior therapy for anxiety disorders: a pragmatic randomized clinical trial. Psychological Medicine, 1-11. 10.1017/S0033291720004316 10.1017/S0033291720004316 PMC9647541 33261700

[13] Brown TA, Barlow DH (2014). Anxiety and Related Disorders Interview Schedule for DSM-5 - Adult and Lifetime Version: Clinician Manual.

[14] Spitzer RL, Kroenke K, Williams JB, Löwe B (2006). A brief measure for assessing generalized anxiety disorder: the GAD-7. Arch Intern Med.

[15] Norton PJ. Group cognitive-behavioral therapy of anxiety: A transdiagnostic treatment manual. New York: Guilford Press; 2012.

[16] Beaton DE, Bombardier C, Guillemin F, Ferraz MB (2000). Guidelines for the process of cross-cultural adaptation of self-report measures. Spine (Phila Pa 1976)..

[17] Wild D, Grove A, Martin M, Eremenco S, McElroy S, Verjee-Lorenz A, Erikson P (2005). Principles of good practice for the translation and cultural adaptation process for patient-reported outcomes (PRO) measures: report of the ISPOR Task Force for Translation and Cultural Adaptation. Value in Health.

[18] Beck AT, Epstein N, Brown G, Steer RA (1988). An inventory for measuring clinical anxiety: psychometric properties. J Consult Clin Psychol.

[19] Freeston MH, Ladouceur R, Thibodeau N, Gagnon F, Rheaume J (1994). L'inventaire d'anxiété de Beck : Propriétés psychométriques d'une traduction française. L'Encephale.

[20] Chambless DL, Caputo GC, Jasin SE, Gracely EJ, Williams C (1985). The Mobility Inventory for Agoraphobia. Behav Res Ther.

[21] Stephenson R, Marchand A, Lavallée M (1997). Validation de l’Inventaire de mobilité pour l’agoraphobie auprès de la population québécoise francophone. Science Et Comportement.

[22] Connor KM, Davidson JR, Churchill LE, Sherwood A, Foa E, Weisler RH (2000). Psychometric properties of the Social Phobia Inventory (SPIN). New self-rating scale Br J Psychiatry.

[23] Radomsky, A.S., Ashbaugh, A.R., Saxe, M.L., Ouimet, A.J., Golden, E.R., Lavoie, S.L., O'Connor, K.P., 2006. Psychometric properties of the French and English versions of the Social Phobia Inventory. Canadian Psychological Association, pp. 354–360. 10.1037/cjbs2006021

[24] Gosselin, P., Dugas, M.-J., Ladouceur, R., Freeston, M.-H., 2001. Évaluation des inquiétudes : validation d’une traduction française du Penn State Worry Questionnaire. L'Encephale 27. 11760697

[25] Meyer TJ, Miller ML, Metzger RL, Borkovec TD (1990). Development and validation of the Penn State Worry Questionnaire. Behav Res Ther.

[26] Arthurs, E., Steele, R.J., Hudson, M., Baron, M., Thombs, B.D., Group, C.C.S.R. (2012). Are scores on English and French versions of the PHQ-9 comparable? An assessment of differential item functioning. PLoS One.

[27] Kroenke K, Spitzer RL, Williams JB (2001). The PHQ-9: validity of a brief depression severity measure. J Gen Intern Med.

[28] Sheehan DV, Harnett-Sheehan K, Raj BA (1996). The measurement of disability. Int Clin Psychopharmacol.

[29] Blais FC, Gendron L, Mimeault V, Morin CM (1997). Evaluation of insomnia: validity of 3 questionnaires. L'Encephale.

[30] Morin C (1993). Insomnia: Psychological Assessment and Management.

[31] Cheng-Hsien L (2016). Confirmatory factor analysis with ordinal data: Comparing robust maximum likelihood and diagonally weighted least squares. Behav Res.

[32] Schreiber JB, Nora A, Stage FK, Barlow EA, King J (2006). Reporting Structural Equation Modeling and Confirmatory Factor Analysis Results: A Review. J Educ Res.

[33] Swank JM, Mullen PR (2017). Evaluating Evidence for Conceptually Related Constructs Using Bivariate Correlations. Meas Eval Couns Dev.

