# Supplementary material for: French adaptation and validation of the Panic Disorder Severity Scale—self-report
Source: BMC Psychiatry. 2022 Jun 27;22:434. doi: 10.1186/s12888-022-03989-x (PMC9235095; doi:10.1186/s12888-022-03989-x)
Supplement: Supplementary file 1 — Additional file 1: Supplementary Table 1. Supplementary Figure 1. Path diagram from confirmatory factor analysis. [file 12888_2022_3989_MOESM1_ESM.docx]

**Supplementary Table**

| PDSS-SR cut-point | Sensitivity | Specificity | Youden index | Positive predictive Value | Negative predictive Value |
| --- | --- | --- | --- | --- | --- |
| 0 | 1.00 | 0.00 | 0.000 | 0.41 | 1.00 |
| 1 | 1.00 | 0.18 | 0.178 | 0.45 | 1.00 |
| 2 | 0.99 | 0.26 | 0.254 | 0.48 | 0.98 |
| 3 | 0.98 | 0.34 | 0.316 | 0.5 | 0.96 |
| 4 | 0.94 | 0.38 | 0.324 | 0.51 | 0.91 |
| 5 | 0.91 | 0.43 | 0.348 | 0.52 | 0.88 |
| 6 | 0.88 | 0.48 | 0.355 | 0.54 | 0.85 |
| 7 | 0.86 | 0.57 | 0.422 | 0.57 | 0.85 |
| 8 | 0.81 | 0.65 | 0.459 | 0.61 | 0.83 |
| 9 | 0.79 | 0.70 | 0.492 | 0.65 | 0.83 |
| 10 | 0.74 | 0.75 | 0.490 | 0.67 | 0.81 |
| 11 | 0.65 | 0.81 | 0.463 | 0.7 | 0.77 |
| 12 | 0.61 | 0.86 | 0.468 | 0.75 | 0.76 |
| 13 | 0.54 | 0.91 | 0.446 | 0.8 | 0.74 |
| 14 | 0.51 | 0.93 | 0.444 | 0.84 | 0.74 |
| 15 | 0.40 | 0.95 | 0.351 | 0.84 | 0.70 |
| 16 | 0.31 | 0.96 | 0.268 | 0.84 | 0.67 |
| 17 | 0.25 | 0.97 | 0.224 | 0.87 | 0.65 |
| 18 | 0.21 | 0.98 | 0.192 | 0.88 | 0.65 |
| 19 | 0.14 | 0.99 | 0.138 | 0.94 | 0.63 |
| 20 | 0.09 | 0.99 | 0.080 | 0.9 | 0.61 |
| 21 | 0.08 | 0.99 | 0.070 | 0.89 | 0.61 |
| 22 | 0.06 | 0.99 | 0.051 | 0.86 | 0.61 |
| 23 | 0.04 | 0.99 | 0.032 | 0.8 | 0.60 |
| 24 | 0.03 | 0.99 | 0.022 | 0.75 | 0.60 |
| 25 | 0.02 | 0.99 | 0.013 | 0.67 | 0.60 |
| 26 | 0.00 | 0.99 | -0.007 | 0.00 | 0.59 |
| 27 | 0.00 | 1.00 | 0.000 | 0.00 | 0.59 |

**
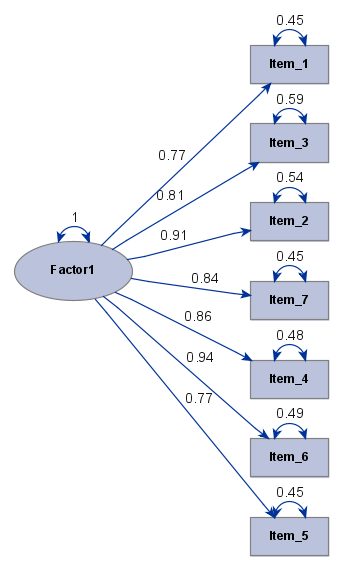
**

**Supplementary Figure. Path diagram from confirmatory factor analysis**
